# Suppression of CHOP Reduces Neuronal Apoptosis and Rescues Cognitive Impairment Induced by Intermittent Hypoxia by Inhibiting Bax and Bak Activation

**DOI:** 10.1155/2021/4090441

**Published:** 2021-08-21

**Authors:** Linhao Xu, Yanli Bi, Yizhou Xu, Yihao Wu, Xiaoxue Du, Yixuan Mou, Jian Chen

**Affiliations:** ^1^Department of Cardiology, Affiliated Hangzhou First People's Hospital, Zhejiang University School of Medicine, Hangzhou 310006, China; ^2^School of Basic Medical Sciences and Forensic Medicine, Hangzhou Medical College, Hangzhou 310053, China; ^3^Key Laboratory of Clinical Cancer Pharmacology and Toxicology Research of Zhejiang Province, Affiliated Hangzhou First People's Hospital, Zhejiang University School of Medicine, Hangzhou 310006, China; ^4^Department of Clinical Laboratorial Examination, Air Force Hangzhou Special Service Recuperation Center Sanatorium Area 3, Hangzhou 310002, China

## Abstract

Our previous study showed that growth arrest- and DNA damage-inducible gene 153 (GAD153/CHOP) plays an important role in intermittent hypoxia- (IH-) induced apoptosis and impaired synaptic plasticity. This study is aimed at determining which signaling pathway is activated to induce CHOP and the role of this protein in mitochondria-dependent apoptosis induced by IH. In the in vivo study, mice were placed in IH chambers for 8 h daily over a period of 2 weeks; the IH chambers had oxygen (O_2_) concentrations that oscillated between 10% and 21%, cycling every 90 s. In the in vitro study, PC12 cells were exposed to 21% O_2_ (normoxia) or 8 IH cycles (25 min at 21% O_2_ and 35 min at 0.1% O_2_ for each cycle). After 2 weeks of IH treatment, we observed that the expression levels of phosphorylated protein kinase-like endoplasmic reticulum kinase (p-PERK), activating transcription factor 4 (ATF-4) and phosphorylated eukaryotic initiation factor 2 alpha (p-elf2*α*), were increased, but the levels of activating transcription factor 6 (ATF-6) and inositol-requiring enzyme 1 (IRE-1) were not increased. GSK2606414, a specific chemical inhibitor of the PERK pathway, reduced the expression of p-PERK, ATF-4, p-elf2*α*, and CHOP and rescued ER structure. In addition, Bax and Bak accumulated in the mitochondria after IH treatment, which induced cytochrome c release and initiated apoptosis. These effects were prevented by GSK2606414 and CHOP shRNA. Finally, the impaired long-term potentiation and long-term spatial memory in the IH group were rescued by GSK2606414. Together, the data from the in vitro and in vivo experiments indicate that IH-induced apoptosis and impaired synaptic plasticity were mediated by the PERK-ATF-4-CHOP pathway. Suppressing PERK-ATF-4-CHOP signaling pathway attenuated mitochondria-dependent apoptosis by reducing the expression of Bax and Bak in mitochondria, which may serve as novel adjunct therapeutic strategy for ameliorating obstructive sleep apnea- (OSA-) induced neurocognitive impairment.

## 1. Introduction

Obstructive sleep apnea (OSA) is a common disorder characterized by repetitive collapse of the pharyngeal airway during sleep, resulting in sleep fragmentation and intermittent hypoxia (IH) [1]. As one major consequence of OSA disease, IH leads to sympathetic nervous system activation and oxidative stress [2], which results in neurocognitive dysfunction. As one harmful consequence induced by chronic IH, apoptotic neuronal cell death is strongly believed to contribute to cognitive impairment in OSA disease which was shown in animal models and clinical studies [3, 4]. According to our previous studies, endoplasmic reticulum (ER) stress-mediated apoptosis was observed in the hippocampus of a mouse IH model [5, 6]; however, the specific mechanism by which IH induction led to ER stress-mediated apoptosis was not fully described.

The ER is an important organelle with multiple functions in which all secretory and integral membrane proteins are folded. When a stressor, such as hypoxia, glucose deprivation, or protein overload from a virus, blocks ER function, the accumulation of unfolded or misfolded proteins in the ER affects cellular functions, and the adaptive unfolded protein response (UPR) is triggered [7]. However, when injury is excessive, the ER stress signal transduction pathways can also induce cell death. According to previous studies, three signaling pathways, including the protein kinase-like endoplasmic reticulum kinase (PERK), activating transcription factor 6 (ATF6), and inositol-requiring enzyme 1 (IRE1) pathways, are all necessary for the activation of growth arrest and DNA damage-inducible gene 153- (CHOP/GAD153-) mediated apoptosis [8].

CHOP is an ER stress-specific transcription factor that causes changes in gene expression that favor apoptosis, including increasing the expression of Bim and decreasing the expression of Bcl-2 [9]. It has been shown that Bim has an important role in mitochondrial membrane potential stability [10]. Upregulated CHOP increases the expression of Bim, which transports the Bax and Bak proteins to the outer membranes of mitochondria under ER stress; then, Bax and Bak increase the permeability of outer mitochondrial membranes and induce cytochrome c release from the mitochondria to the cytoplasm, finally resulting in caspase-dependent apoptosis [11, 12].

This study is mainly aimed at investigating the specific mechanism of CHOP expression induction and the role of CHOP in neuronal apoptosis in the hippocampus of an IH mouse model. Therefore, we used GSK2606414, a specific chemical inhibitor of the PERK pathway, to suppress the expression of CHOP in an animal model. In an in vitro study, CHOP was knocked down by lentivirus transfection. Thus, in this study, the effects of CHOP on apoptosis in IH models were examined.

## 2. Materials and Methods

### 2.1. Animals

Six-week-old male C57BL/6J mice weighing 20–22 g were housed under the following standard conditions: 22°C, a 12 h light/12 h dark cycle, and standard food and water provided ad libitum. All procedures performed in this study were in accordance with the National Institutes of Health Guide for the Care and Use of Laboratory Animals. Permission for the performance of the animal studies was granted by the Ethics Committee of Zhejiang University.

### 2.2. Mouse Model of Chronic IH

The protocol was based on well-reported rodent models of sleep apnea as described in our previous studies and by other researchers [5, 6, 13]. Ordinary cages were placed in computer-controlled ventilation chambers (OxyCycler model A48XOV, Redfield, USA) to achieve IH exposure in the animals. The concentration of oxygen (O_2_) was maintained between 10% and 21%, cycling every 90 s for 8 h (from 8 : 00 AM to 4 : 00 PM). The control animals were exposed to alternating periods of room air in identical chambers. The IH treatment lasted for 2, 3, 7, or 14 days.

### 2.3. Material

GSK2606414 was purchased from AdooQ Bioscience (Irvine, CA, USA). Hydroxypropylmethyl cellulose and Tween-80 were obtained from Sigma-Aldrich (St. Louis, MO, USA). Antibodies against p-PERK (cat: 3179), activating transcription factor 4 (ATF-4, cat: 11815), phosphorylated eukaryotic initiation factor 2 alpha (p-elf2*α*, cat: 3398), cleaved caspase-3 (cat: 9661), Bak (cat: 6947), cytochrome c (cat: 11940), and COX IV (cat: 4884) were purchased from Cell Signaling Technology (Danvers, Beverly, MA). Antibodies against Bax (cat: ab32503) and *β*-actin (cat: ab8226) were purchased from Abcam (Cambridge, USA). Antibodies against Bim (cat: DF6093), phosphor-ATF-6 (cat: DF4507), phosphor-IRE1 (cat: AF7150), and CHOP (cat: DF6025) were purchased from Affinity Biosciences (Cincinnati, OH, USA). NE-PER™ Nuclear and Cytoplasmic Extraction Reagents (cat: 78833), Mitochondria Isolation Kit for Tissue (cat: 89801), and Mitochondria Isolation Kit for Tissue for Cells (cat: 78510) were purchased from Thermo Fisher Scientific (Waltham, MA, USA).

### 2.4. Drug Treatment

GSK2606414 was suspended in vehicle (0.5% hydroxypropylmethyl cellulose +0.1% Tween-80 in H_2_O at pH 4.0) at a concentration of 5 mg/ml. GSK2606414 was given by oral gavage once daily at a dose of 50 mg/kg body weight after 1 week of exposure to IH. The mice were randomly assigned into the following four groups: the vehicle-treated control group (Control), the GSK2606414-treated control group (GSK2606414), the vehicle-treated IH group (IH), and the GSK2606414-treated IH group (IH + GSK2606414).

### 2.5. Behavioral Studies

After 2 weeks of IH exposure, the behavioral experiments were performed between 8 : 00 AM and 4 : 00 PM and were recorded using an online video tracking system (ANY-maze, Global Biotech Inc., Shanghai, China). Three behavioral experiments were conducted. Exploratory behavior in the open-field test was used to measure anxiety levels, as described in a previous study [14]. A delay-dependent one-trial object recognition task was performed to evaluate hippocampus-dependent long-term recognition memory, and the Morris water maze test was employed to assess hippocampus-dependent spatial memory [15]. A detailed description of these procedures is available in the Supplementary Information.

### 2.6. LTP Measurement

The mice were sacrificed by decapitation, and the brains were immediately removed and cut into two halves in the sagittal plane. They were then immersed in ice-cold artificial cerebrospinal fluid (ACSF). The ACSF contained 125 mM NaCl, 2.0 mM KCl, 1.2 mM MgSO_4_, 2.5 mM CaCl_2_, 1.2 mM KH_2_PO_4_, 10 mM glucose, and 26 mM NaHCO_3_. Then, the brain was sagittally sliced at 300 *μ*m using a vibrating microtome (In-tegraslice 7550MM; Campden Instruments Ltd.). Then, those slices were incubated in warm (32-34°C), oxygenated (95%O_2_/5%CO_2_) ACSF for at least 1 hour. Afterward, these slices were transferred to submersion-type P210A probes (Alpha Med Sciences Co., Ltd., Tokyo, Japan) perfused constantly with ACSF at a rate of 1.0-1.3 ml/min. The temperature of the ACSF in this recording probe was maintained at 34 ± 1°C by a heat-exchanger. A planar multielectrode array recording system (MED64 system; Alpha Med Sciences Co., Ltd.) was employed to record fEPSP for LTP measurement. fEPSPs were then recorded from the dendritic layer of CA1 neurons while providing single-pulsed electrical stimulation (0.017 Hz, stimulus duration 100 *μ*s) to the Schaffer collateral pathway. According to our previous studies [5, 6], we chose a stimulation intensity that evoked the fEPSP with a magnitude of 30–40% of the maximum response. After allowing a stable baseline of 30 min, a single 1 s train of 100 Hz stimulation (10 ms inter-stimulus interval, stimulus duration 100 *μ*s) was applied to induce LTP, which was quantified as the percentage increase of the average fEPSP amplitude recorded in the 50 to 60 min interval after high-frequency stimulation compared with the baseline value.

### 2.7. Transmission Electron Microscopy

Transmission electron microscopy was used to investigate the ultrastructural changes in the endoplasmic reticulum and mitochondria. The entire hippocampal region (three mice for each group) was dissected and diced into 1 mm^3^ piece and then fixed using 2.5% glutaric dialdehyde for 4 h. The pieces were postfixed in 2% osmium tetroxide for 30 min, dehydrated in graded alcohol, transferred to propylene oxide, and gradually embedded in blocks of Epon 812 resin for 2 days at 60°C. Eighty-nanometer sections were collected using a diamond histoknife (Diatome Ltd, Nidau, Switzerland) on an UltraCut E Microtome (Leica Inc., Buffalo Grove, IL United States) and then mounted on a copper mesh and stained with uranyl acetate and lead nitrate. The ultrastructural changes were identified by a transmission electron microscope (Hitachi H-7700, Tokyo, Japan).

### 2.8. Cell Culture and Lentiviral Transfection

PC12 cells (rat phaeochromocytoma cells) were cultured in Dulbecco's Modified Eagle's Medium (DMEM) supplemented with 10% fetal bovine serum, 100 U/mL streptomycin, and 100 U/mL penicillin in a humidified chamber maintained with 5% CO_2_ and 21% O_2_ at 37°C. The lentivirus vector was purchased from GenePharma Bio-Tech Company (Shanghai, China). The sequence of the siRNA targeting rat CHOP was 5′-GAGGAAGATCAAGGAAGAACT-3′, and this siRNA efficiently suppressed the expression of CHOP mRNA (Supplementary Figure 1). A scrambled control siRNA (5′-TTCTCCGAACGTGTCACGT-3′) was used as a control. The detailed procedure of constructing the rat CHOP shRNA lentiviral expression vector is provided in the Supplementary Information. The titers of lentiviral particles were approximately 5 × 10^8^ units/ml. After the cells reached 50–60% confluence, the virus was added, and the cells were transfected for 48 h. qRT-PCR was used to assess the effect of lentivirus on suppressing target mRNA expression, which is described in the Supplementary Information.

### 2.9. Chronic Intermittent Hypoxia Cultured PC12 Cells

After transfection, PC12 cells were either exposed to normoxia (21% O_2_, 5% CO_2_, and balance N_2_) or to intermittent hypoxia (consisting of 8 episodic cycles of normoxia (21% O_2_, 5% CO_2_, and balance N_2_) for 25 min and hypoxia (0.1% O_2_, 5% CO_2_, and balance N_2_) for 35 min) using a custom-designed, computer-controlled incubation chamber attached to an external O_2_/CO_2_ computer-driven controller (BioSpherix, Redfield, NY) according to a previous study (Shan et al., 2007). Chamber O_2_, N_2_, and CO_2_ levels were continuously monitored and adjusted according to the desired programmed profile. The cells were divided into the following four groups: the control group (normoxia), the control+siRNA CHOP group, the IH group, and the IH + siRNA CHOP group.

### 2.10. Cytoplasmic and Mitochondrial Protein Extraction

Cytoplasmic protein was extracted from hippocampal tissue and PC12 cells using NE-PER™ Nuclear and Cytoplasmic Extraction Reagents (Thermo Fisher Scientific, Waltham, MA, USA). First, hippocampal tissue or harvested PC12 cells were washed with PBS and then centrifuged at 500 × g for 5 minutes. The supernatant was discarded, and the tissue or cell pellet was homogenized with the CER I reagent from the kit. Then, the tube was shaken vigorously for 15 seconds to fully suspend the tissue or cell pellet and incubated on ice for 10 minutes. Next, the CER II reagent from the kit was added and incubated on ice for 1 minute. After shaking for 5 seconds, the tube was centrifuged at 16,000 × g. Finally, the supernatant (i.e., the cytoplasmic extract) was immediately transferred to a clean tube, and routine immunoblotting was used to detect protein expression.

Mitochondrial protein was extracted using a Mitochondria Isolation Kit (Thermo Fisher Scientific, Waltham, MA, USA). Hippocampal tissue or harvested PC12 cells were washed with PBS and centrifuged at 850 × g for 2 minutes, and the supernatant was discarded. Second, mitochondria isolation reagent A was added to the suspended pellet and incubated for 2 minutes. After vortexing for 5 seconds, mitochondria isolation reagent B was added, and the tube was incubated on ice for 5 minutes. Then, mitochondria isolation reagent C was added, and the tube was inverted several times to mix. Next, the tube was centrifuged at 700 × g for 10 minutes at 4°C. The supernatant was transferred to a new tube and centrifuged at 12,000 × g for 15 minutes at 4°C. Finally, the pellet (i.e., the isolated mitochondria) was used to detect protein expression.

### 2.11. Western Blot Analysis

Total proteins were extracted from hippocampal tissue and PC12 cells by ice-cold radioimmunoprecipitation assay buffer containing 1 mM PMSF according to the manufacturer's instructions (Beyotime Biotechnology, Shanghai, China). The concentrations of cytoplasmic protein, mitochondrial protein, and total protein were assessed by the BCA Protein Assay Reagent Kit (Thermo Fisher Scientific, Waltham, MA, USA). Thirty micrograms of protein was separated by SDS-PAGE and transferred to nitrocellulose filter membranes (Millipore Corporation) at 100 V for 1 h by the wet transfer method. The membranes were blocked with 5% nonfat dry milk at 37°C for 1 h and then incubated with the relevant primary antibodies at 4°C overnight. The expression levels of p-PERK, p-elf2*α*, ATF-4, ATF-6, IRE-1, Bim, and CHOP were detected in the total protein fraction, and the expression levels of Bax, Bak, and cytochrome C were evaluated in the cytoplasmic and mitochondrial protein fractions. Then, the membranes were incubated with horseradish peroxidase-conjugated secondary antibodies, and the immunoreactive bands were visualized using an ECL kit (Thermo Fisher Scientific, Waltham, MA, USA). Equal target protein expression was confirmed by normalization to *β*-actin or COX IV.

### 2.12. Isolation of Mitochondria and Measurement of Mitochondrial Function

Mitochondria were isolated from hippocampus using the discontinuous Percoll density gradient method [16]. The mitochondrial membrane potential was assessed using the lipophilic cationic carbocyanine probe JC-1 [17]. More detailed information is included in the Supplementary Information.

### 2.13. Data Analysis

The data are expressed as the mean ± standard deviation. Significant differences were determined by one-way ANOVA followed by Tukey's test as the post hoc test for multiple groups. Unpaired Student's *t*-tests were used to compare between two different groups. A *P* value of < 0.05 was regarded as statistically significant.

## 3. Results

### 3.1. The PERK Pathway but Not the ATF-6 or IRE-1 Pathway Was Activated after 2 Weeks of IH Exposure

**Figure 1:**
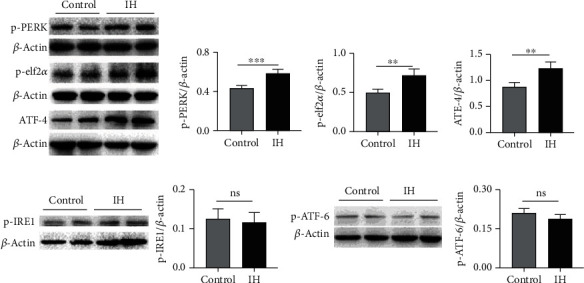
The PERK pathway but not the ATF-6 or IRE-1 pathway was activated. (a) The expression levels of p-PERK, p-elf2*α*, and ATF-4 were increased in the hippocampus after 2 weeks of IH exposure (*n* = 4). The expression of p-IRE1 (b) and p-ATF-6 (c) was not altered (*n* = 4). ^∗^*P* < 0.05; ^∗∗^*P* < 0.01; ns: not significant.

As discussed above, three main pathways are involved in ER stress-induced CHOP expression, which was increased after 2 weeks of IH treatment; therefore, in this study, we first investigated which specific pathway plays an important role in regulating the expression of CHOP. A number of proteins involved in the PERK pathways were assessed. As shown in Figure 1(a), compared with that in the control group, the expression of p-PERK, p-elf2*α*, and ATF-4 was significantly increased in the IH group; however, the expression of ATF-6 and p-IRE1 did not increase after 2 weeks of IH exposure.

### 3.2. GSK2606414 Inhibits PERK Pathway and Rescues the ER Morphology

**Figure 2:**
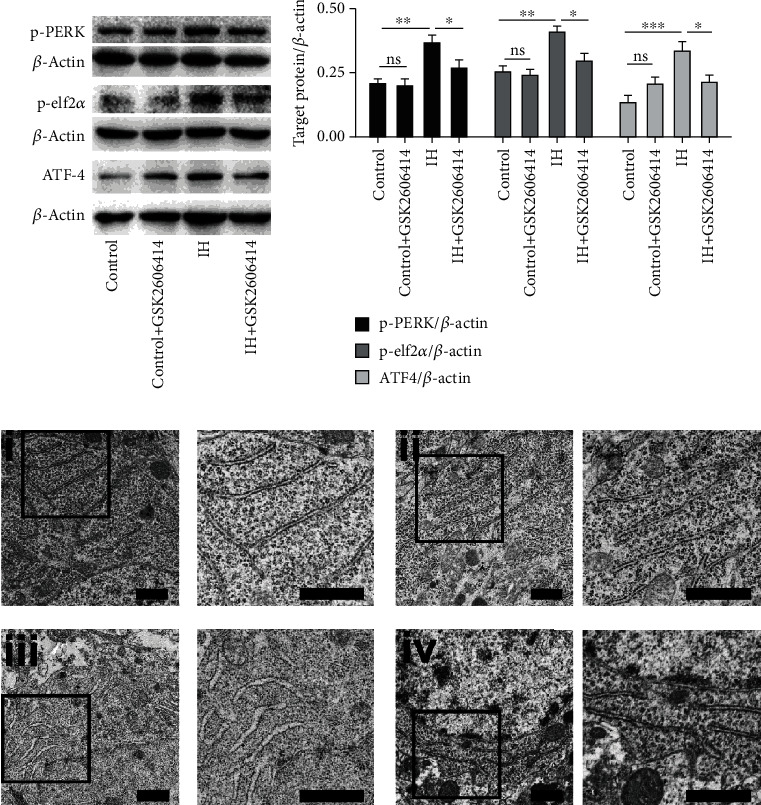
GSK2606414 inhibited the PERK pathway and rescued ER morphology. (a) Upregulation of p-PERK, p-elf2*α*, and ATF-4 was reduced by GSK2606414 administration. (b) The pooled data from three mice in each group are summarized. (c) Clear and intact ER ultrastructure (indicated by arrowheads) was observed in the control group (i) and the GSK2606414 group (ii). However, 14 days of IH caused ER swelling and distortion, suggesting protein aggregation in the lumen (iii). GSK2606414 attenuated the injury to ER structure induced by IH treatment (iv). The inset in each picture is enlarged and displayed on the right. ^∗^*P* < 0.05; ^∗∗^*P* < 0.01; ns: not significant. Scale bars: 0.5 *μ*m.

To confirm that PERK indeed participated in ER stress activation, GSK2606414, a PERK inhibitor, was administered by oral gavage after 1 week of IH treatment. As shown in Figure 2(a), the levels of three proteins (p-PERK, p-elf2*α*, and ATF-4) in the PERK pathway were significantly reduced when the animals were treated with the PERK inhibitor GSK2606414 after 1 week of IH treatment (*P* < 0.05 compared with the IH group).

Furthermore, the effect of GSK2606414 on the morphology of the ER in the hippocampus was obtained by ultrastructural analysis. Electron microscopy revealed intact and parallel rows of rough ER in the control group and the GSK2606414 group (Figures 2(c)i and 2(c)ii); however, the ER in the IH-treated group was not only swollen but also distorted and lost the typical parallel arrangement, which was consistent with our previous work (Figure 2(c)iii). GSK2606414 treatment prevented the distorted appearance of the ER in hippocampal CA1 neurons (Figure 2(c)iv).

### 3.3. GSK2606414 Reduces the Activation of Caspase-3 via Mitochondria-Dependent Apoptosis

**Figure 3:**
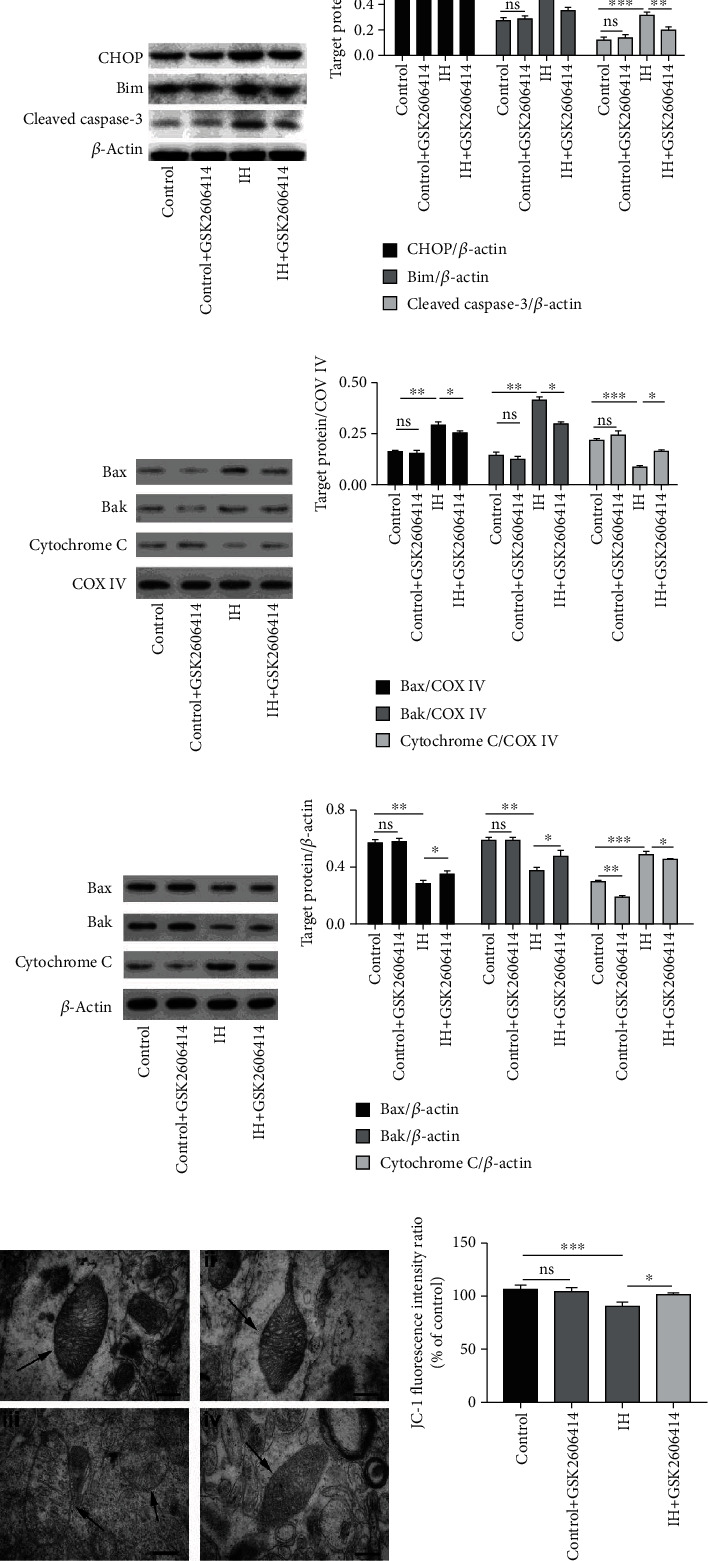
GSK2606414 reduced the activation of caspase-3 via mitochondria-dependent apoptosis. (a) Representative western blots showed that GSK2606414 reduced the expression of CHOP, Bim, and cleaved caspase-3 in hippocampal tissue. The pooled data from three mice for each group are summarized in (b). (c) The western blot results showed that the expression of Bax and Bak in the mitochondria was obviously increased and that this effect was accompanied by a reduction in cytochrome c. These effects were prevented by the administration of GSK2606414. (d) Statistical data from three animals for each group are summarized. (e) Representative western blots showing that the cytoplasmic expression of Bax and Bak was obviously reduced and that of cytochrome C was significantly increased. GSK2606414 increased the expression of Bax and Bak and reduced cytochrome C. (f) Statistical data from three animals for each group are summarized. (g) The morphology of mitochondria in the hippocampal CA1 region in the (i) control group, (ii) GSK2606414 group, (iii) IH group, and (iv) IH + GSK2606414 group. In the control and GSK2606414 groups, intact mitochondria (indicated by arrowheads) with clear cristae were found. Much fewer cristae were found in the mitochondria from the IH group. GSK2606414 treatment preserved more intact mitochondria with discernable cristae. Scale bar: 0.25 *μ*m. (h) The mitochondrial membrane potential (JC-1 fluorescence intensity ratio) was impaired by IH treatment and rescued by GSK2606414 treatment. ^∗^*P* < 0.05; ^∗∗^*P* < 0.01; ns: not significant.

According to a previous study, IH treatment induces apoptosis by activating cleaved caspase-3; however, the full mechanism is still elusive. Here, we found that the increases in the expression of CHOP and Bim and the activation of cleaved caspase-3 were both suppressed by GSK2606414 (Figure 3(a)).

It is well known that CHOP and Bim are both involved in the mitochondria-mediated apoptotic pathway. The expression of three marker proteins, Bax, Bak, and cytochrome c, was measured in the cytoplasm and mitochondria. The results showed that the expression of Bax and Bak was increased in mitochondria and decreased in the cytoplasm after IH, which suggested that these two proteins were translocated (Figures 3(c)–3(f)). Moreover, we observed that cytochrome c was released into the cytoplasm after 14 days of IH treatment. However, the expression of Bax and Bak was reduced by blocking the PERK pathway with GSK2606414, which also increased cytochrome c expression in mitochondria (Figures 3(c)–3(f)).

Furthermore, the mitochondria in CA1 neurons of the control group (Figure 3(g)i) and GSK2606414 group (Figure 3(g)ii) displayed an intact structure with clear cristae; however, most of the mitochondrial cristae disappeared in the IH group (Figure 3(g)iii). After GSK2606414 treatment, the cristae were more intact and easier to observe (Figure 3(g)iv). Finally, a significant decline in the ratio of JC-1 fluorescence intensity was observed in the IH group, and this effect was prevented by GSK2606414 treatment (Figure 3(h)).

### 3.4. Knockdown of CHOP Could Rescue IH-Induced Apoptosis via Mitochondria Pathway

**Figure 4:**
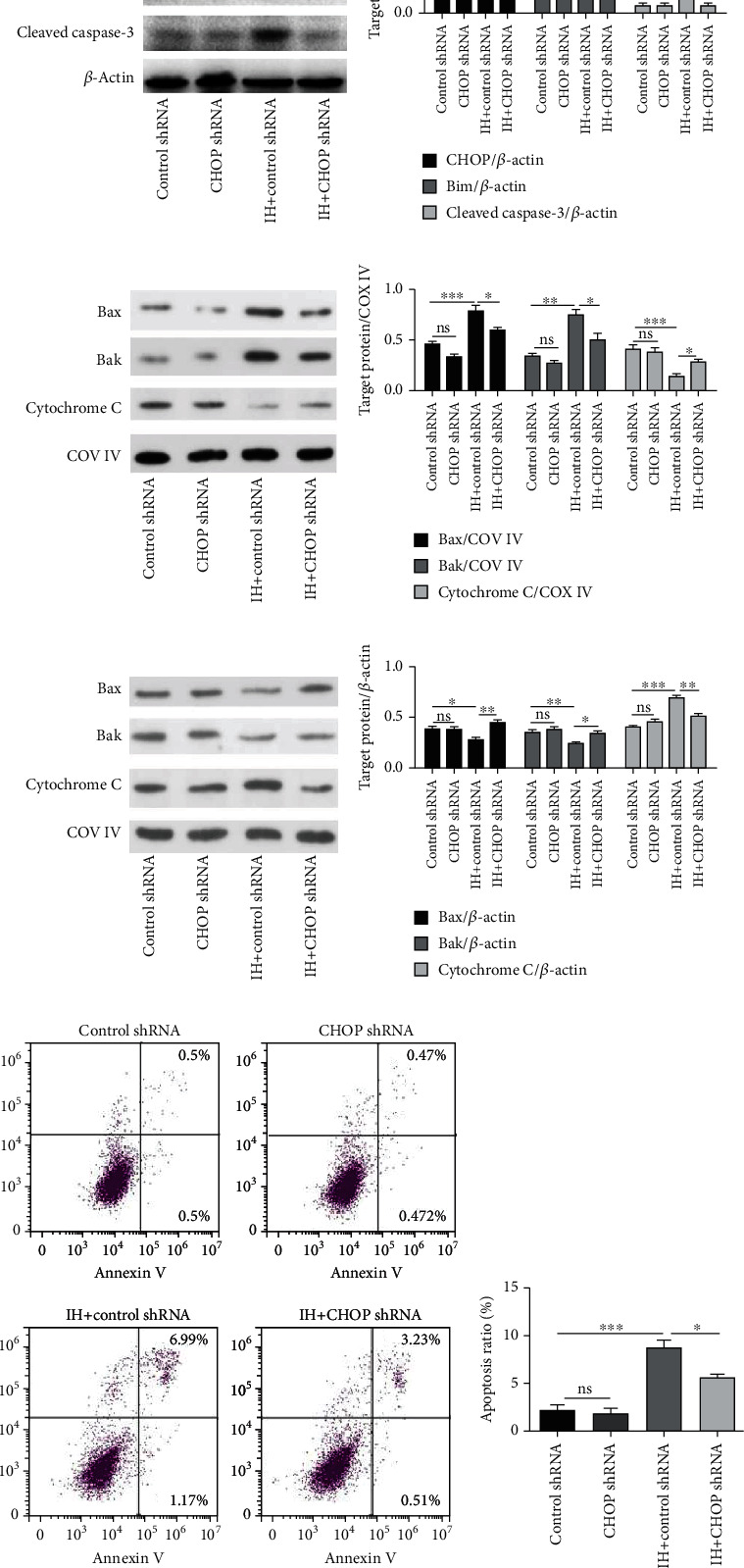
Knockdown of CHOP rescued IH-induced apoptosis via the mitochondrial pathway. (a) Inhibition of CHOP reduced the expression of Bim and cleaved caspase-3, which was increased after IH treatment in the PC12 cell line. (b) The pooled data from each group are summarized (*n* = 3). (c) The western blot results showed that CHOP shRNA decreased the expression of Bax and Bak and elevated the expression of cytochrome C in mitochondria. Statistical data from three animals for each group are summarized in (d) (*n* = 3). (e) Representative western blots showing that the reduced expression of Bax and Bak and increased expression of cytochrome C induced by IH treatment were prevented by CHOP shRNA. (f) The pooled data from each group are summarized (*n* = 3). (g) Annexin V-FITC/PI staining measured by flow cytometry revealed that IH significantly increased the apoptotic cell ratio, which was decreased by CHOP shRNA. (h) Quantification of the results of flow cytometry. ^∗^*P* < 0.05; ^∗∗^*P* < 0.01; ^∗∗∗^*P* < 0.001; ns: not significant.

To confirm the critical role of CHOP in IH-induced apoptosis, PC12 cells were transfected with lentivirus to suppress the expression of CHOP; and after 48 h of transfection, the expression level of CHOP mRNA was significantly reduced (Supplementary Figure 1). As shown in Figures 4(a) and 4(b), we observed that the increased expression of CHOP protein induced by IH treatment was indeed decreased in the IH + CHOP shRNA group and that suppression of CHOP expression also reduced the expression of Bim and cleaved caspase-3. Furthermore, CHOP shRNA reduced the expression of Bax and Bak in mitochondria and the expression of cytochrome c in the cytoplasm (Figures 4(c)–4(f)). Finally, according to the flow cytometry results (Figures 4(g) and 4(h)), the percentages of apoptotic cells in the control shRNA group and the CHOP shRNA group were approximately 2.23 ± 1.12% and 1.93 ± 1.00%, respectively. However, the apoptotic ratio in the IH + control shRNA group was 8.84 ± 1.41%, which was significantly higher than that in the IH + CHOP shRNA group (5.64 ± 0.66%).

### 3.5. The Impairment of Hippocampal Synaptic Plasticity Induced by IH Was Rescued by GSK2606414

**Figure 5:**
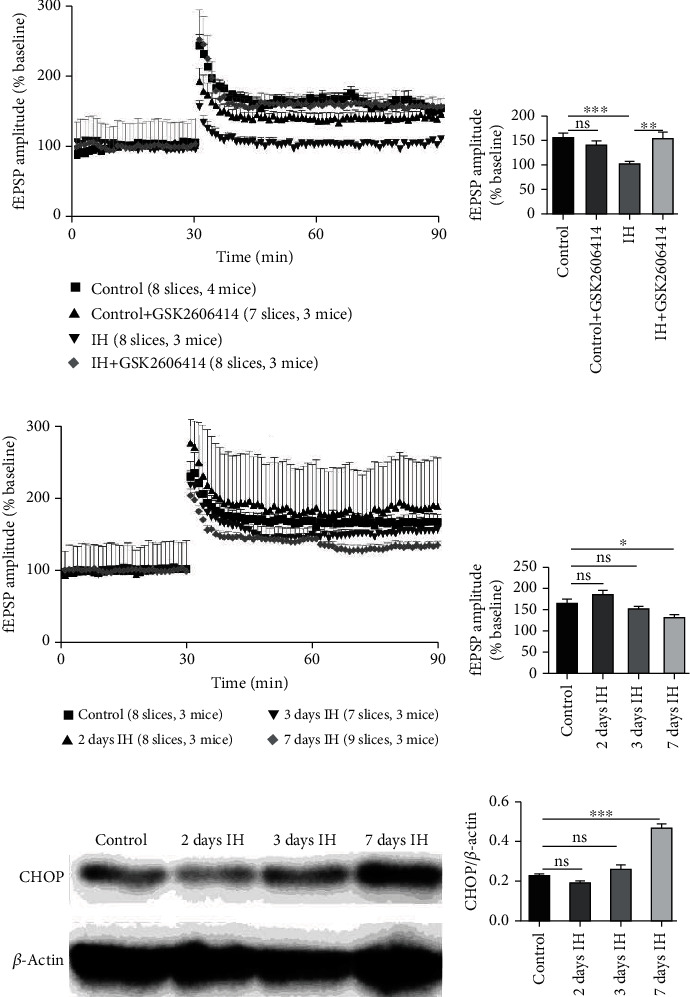
The impairment of hippocampal synaptic plasticity was induced by chronic IH treatment (7 and 14 days) which was associated with the increased expression of CHOP. GSK2606414 could rescue this impairment. (a) GSK2606414 prevented chronic IH-induced impairment in hippocampal LTP. (b) Quantification of the results shown in A. (c) hippocampal synaptic plasticity was not impaired after 2 or 3 days of IH treatment. (d) Quantification of the results shown in C. (e) Representative western blots showed that the expression of CHOP was not increased in hippocampal tissue after 2 or 3 days of IH exposure. The pooled data from three mice for each group are summarized in (f). ^∗^*P* < 0.05; ^∗∗^*P* < 0.01; ^∗∗∗^*P* < 0.001; ns: not significant.

Then, we investigated whether the PERK inhibitor GSK2606414 could prevent chronic IH-induced impairment in hippocampal LTP. As shown in Figures 5(a) and 5(b), the magnitude of LTP was 157.04 ± 4.3% in the control group, while GSK2606414 itself did not have any effect on the potentiation of fEPSPs. On the other hand, the magnitude of LTP was reduced after 14 days of IH treatment (104.61 ± 4.02, *P* < 0.05 compared with the normoxia group) and was significantly restored by administration of GSK2606414 (156.67 ± 11.42, *P* < 0.05 compared with IH alone; *P* > 0.05 compared with the control group and the control+GSK2606414 group).

In order to investigate the effect of short-duration IH treatment on hippocampal synaptic plasticity, the expression of CHOP and LTP after 2 and 3 days IH treatment was detected. The results showed that the expression of CHOP was not increased after 2 or 3 days of IH treatment and increased after 1 week of IH treatment (Figures 5(c) and 5(d)). Meanwhile, LTP magnitude was not altered after two and three days of exposure to IH and reduced after 1 week of IH treatment (Figures 5(e) and 5(f)).

### 3.6. GSK2606414 Rescues IH-Induced Memory Impairment

Finally, two different behavioral experiments were performed, including the one-trial object recognition task for recognition memory and the water maze test, to assess the effect of GSK2606414 on IH-induced memory impairment. During the IH treatment period, we observed that body weight was reduced after IH treatment; however, GSK2606414 did not affect body weight (Supplementary Figure 2).

**Figure 6:**
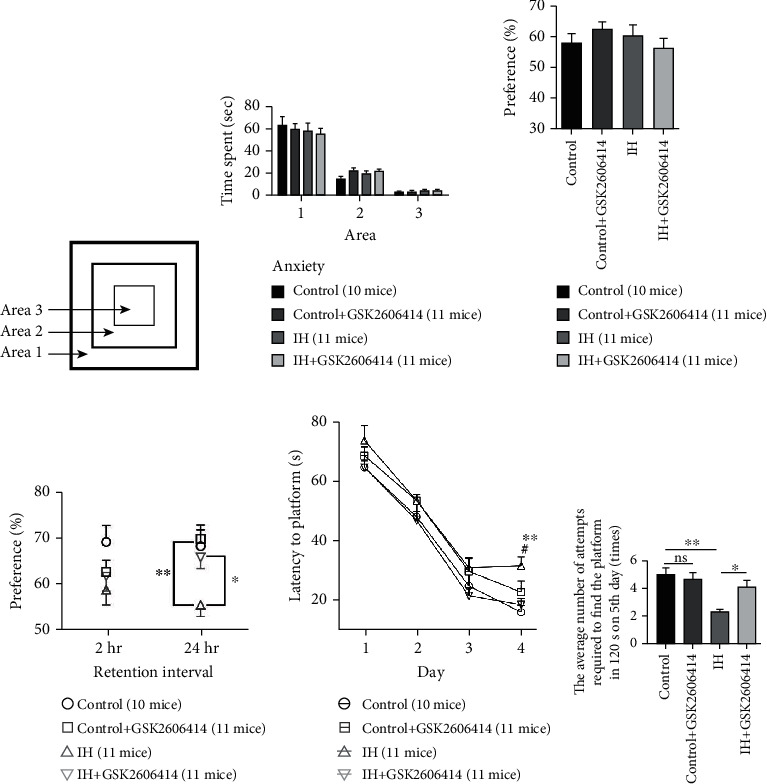
GSK2606414 rescued IH-induced memory impairment. (a) Anxiety levels were assessed using the open-field test. Three concentric areas of the open arena were defined. (b) The amounts of time animals spent in different areas did not differ among groups. (c) The animals in the different groups showed no preference for one object over another identical object during the training phase. (d) The percentage of time spent exploring the novel object was significantly reduced in the IH group during the trial phase 24 h after training. The higher preference for the novel object was restored by GSK2606414. ^∗^*P* < 0.05; ^∗∗^*P* < 0.01. (e) The escape latencies were increased in the IH group on the fourth day and were reduced by GSK2606414. ^∗∗^*P* < 0.01 control vs. IH; ^#^*P* < 0.05 IH vs. IH + GSK2606414. (f) IH increased the number of attempts required to find the platform, and this effect was reduced by GSK2606414. ^∗^*P* < 0.05; ^∗∗^*P* < 0.01; ns: not significant.

Before the one-trial object recognition task, the animals were allowed to move freely in the open field, since the emotional status of the animals may affect their performance in the memory test. Usually, the amount of time spent at the edge of the field is greater than that spent in the central area of the field (Figure 6(a)). As shown in Figure 6(b), the amounts of time that animals in the different groups spent in the three different concentric areas did not differ significantly, suggesting that their anxiety levels were not affected by IH treatment or GSK2606414 administration.

During the training phase, there was no preference for one object over another identical object among the four different groups (Figure 6(c)). However, in the test trial, it was shown that there was a clear decline in preference for exploring the novel object after 24 h of retention in the IH group, and this effect was partially rescued by GSK2606414 treatment (Figure 6(d)).

Furthermore, hippocampus-dependent spatial memory was assessed by the Morris water maze test. As shown in Figure 6(e), there were no significant differences in escape latency among the four groups from the first to the third day of the experiment (*P* > 0.05). However, on the fourth day, the escape latency was much higher in the IH group than in the control group and the control+GSK2606414 group (*P* < 0.01). Moreover, the mice in the IH + GSK2606414 group spent significantly less time searching for the platform than the mice in the IH group. Finally, IH increased the number of attempts required to find the platform, and this effect was reduced by GSK2606414 (Figure 6(f)).

### 3.7. Discussion

Our previous study showed that ER stress was activated in the hippocampus under chronic IH and played an important role in apoptosis [5]; however, the specific mechanism was not fully described. Meanwhile, the most commonly used methods in the clinical treatment of OSA are surgery and continuous positive airway pressure (CPAP) for now; however, each of these two methods has their own shortcomings and it is not apparently beneficial effect on cognitive impairment [18, 19]. Therefore, there is an urgency in seeking a new therapeutic approach, such as drug therapy. However, there have only been a few studies up to now. Therefore, it is worth investigating the molecular mechanisms of apoptosis in OSA.

As discussed above, ER stress is activated under hypoxic conditions. Although it is difficult to provide the fully underlying mechanism of ER stress activation induced by IH, we believe that IH-induced oxidative stress could play a critical role. According to our previous study [5], we had demonstrated that IH could increase reactive oxygen species (ROS) production in the mitochondria via decreasing complex I activity, limiting ROS production by the antioxidant N-acetylcysteine (NAC) could suppress the expression of CHOP which was consistent with other studies [20]. When ER stress is triggered, Grp78 expression is increased, and it dissociates from PERK, IRE1, and ATF-6 to bind with unfolded or misfolded proteins. Our previous work showed that CHOP expression was indeed increased after 2 weeks of exposure to IH; however, the PERK, IRE1, and ATF-6 pathways are all capable of inducing the expression of CHOP [21]. First, PERK phosphorylates elf2*α*, which leads to the regulation of the translation of several genes. The most studied of these genes is ATF-4, which promotes CHOP transcription. Second, activated ATF6 moves to the nucleus and induces the expression of genes with an ER stress response element in their promoter, including CHOP. Finally, the endonuclease activity of IRE1 induced by ATF-6 also activates the expression of CHOP [22]. Our results showed that the expression levels of p-PERK, p-elf2, and ATF-4 were all enhanced after IH treatment; however, the expression levels of p-IRE1 and active ATF-6 were not elevated, suggesting that the IRE1 and ATF6 pathways are probably not involved in this process. Therefore, the increased CHOP expression in our model was due to the activation of the PERK pathway but not the IRE1 or ATF6 pathway. Although the specific mechanism was not classified in our study, previous literature has demonstrated that IRE1 and ATF-6 signaling pathways are inhibited under long-term ER stress, while PERK/eukaryotic translation initiation factor-2 alpha (eukaryotic) is activated continuously [23]. However, a previous study reported that IH exposure decreases rather than increases ER stress markers [24]. This disparity could be due to the duration of IH treatment in this study was only 3 days which is quite different from our model. Meanwhile, the results in the present study indeed showed that the expression of CHOP was not increased after 2 or 3 days of IH treatment, however, increased CHOP expression was observed after 7 and 14 days of IH exposure. In fact, from our previous study, we observe ER stress activation in the hippocampus after 7 days and 14 days of IH treatment [5], which is consistent with this work, since the expression level of protein in ER stress is dynamic. According to previous literature, the PERK-elf2-ATF-4 arm is activated to induce apoptosis after 6 weeks of IH treatment [25].

To confirm that the PERK pathway indeed participated in ER stress activation, GSK2606414, a PERK inhibitor, was used to suppress the PERK pathway, and multiple experiments were performed. First, GSK2606414 reversed the ER swelling and distortion induced by IH exposure and reduced the expression of p-PERK, p-elf2, and ATF-4. Second, the expression of cleaved caspase-3 was reduced by GSK2606414, which suggested that GSK2606414 has a beneficial effect by suppressing apoptosis induced by IH treatment. Third, the impairment of long-term synaptic plasticity in the hippocampus induced by chronic IH exposure was partially rescued by GSK2606414. Finally, the long-term spatial memory and recognition memory were improved by GSK2606414 according to the results of behavioral experiments. Therefore, these data suggest that chronic IH-induced memory impairment is mediated by the PERK pathway. However, the interesting is that the magnitude of tetanus-evoked LTP tended to be higher than that of the normoxia group and returned to baseline level after 3 days of IH exposure; it suggested that there is a biphasic response to different IH treatments on the magnitude of LTP. Although there was no direct evidence to classify the underlying mechanism, it might be associated with increased firing activity. Since in other studies, we found that CA1 pyramidal neurons were tended to increase their firing activities during short-term (2 and 3 days) exposure to IH monitored by implanting microwire electrode recording array (data not shown).

In addition, a previous study showed that after IH exposure, the number of apoptotic neurons in the hippocampal CA1 region of CHOP knockout mice was significantly lower than that of wild-type mice [26]; however, the specific signaling pathway was not described. CHOP induces apoptosis through a variety of downstream signal transduction pathways, including autophagy-dependent apoptosis and mitochondria-dependent apoptosis [8]. In our present study, it was observed that mitochondrial structure was damaged, and mitochondrial membrane potential was decreased after IH exposure; moreover, GSK2606414 indeed rescued mitochondrial morphology and increased mitochondrial membrane potential. Thus, we hypothesized that the apoptosis induced by CHOP was associated with the mitochondrial pathway.

It is well known that loss of mitochondrial membrane potential is one consequence of mitochondrial outer membrane pore (MOMP) opening, which is controlled by Bcl-2 proteins, including Bax and Bak [11, 27]. Normally, Bax and Bak are constantly shuttled between the mitochondria and the cytosol. In healthy cells, a portion of Bak and the majority of Bax molecules are located in the cytosol [28]; however, under ER stress, Bak and Bax shuttling into the cytosol stops, and they consequently accumulate on the mitochondria. In this process, Bax and Bak insert into the outer mitochondrial membrane as oligomers to form MOMP [29]. Then, MOMP opening results in the release of cytochrome c into the cytosol, which initiates the activation of caspase-3 to induce an apoptotic program [29]. Our results showed that suppression of CHOP by both GSK2606414 and lentivirus transfection decreased Bax and Bak levels in mitochondria and cytochrome c levels in the cytosol, and these effects were accompanied by suppression of caspase-3 activation. This suggests that IH-induced apoptosis is dependent on the mitochondrial pathway. Although the mechanism by which CHOP transports Bax and Bak proteins into mitochondria has not been investigated in our model, previous works have confirmed that CHOP directly induces the transcription of Bim, which has an important role in transporting Bax and Bak to the mitochondrial outer membrane [11, 30]. According to a previous study, Bim, a BH3-only protein, has high affinities for binding and activating Bak or Bax; when Bak or Bax is activated and oligomerizes in the mitochondrial outer membrane, the cell is committed to cell death [31]. In the present study, Bim expression was also indeed increased after IH treatment and decreased when the expression of CHOP was suppressed, suggesting that CHOP induced mitochondrial apoptosis by increasing Bim expression.

## 4. Conclusion

The present study reveals that apoptosis induced by IH treatment was mainly mediated by the PERK-ATF-4-CHOP pathway; suppression of CHOP expression had a beneficial effect by rectifying defects at the cellular and behavioral levels, while inhibition of CHOP reduced the expression of Bim and the level of apoptosis by decreasing the accumulation of Bax and Bak in mitochondria.

## Data Availability

All the data used to support the findings of this study are included within the article.

## Additional Points

*Author Statements*. The manuscript is original, has not been submitted to or is not under consideration by another publication, has not been previously published in any language or any form, and contains no disclosure of confidential information or authorship/patent application/funding source disputations.

## Supplementary Materials

Supplementary Materials Supplementary Figure 1: the expression level of CHOP mRNA was significantly decreased after transfection with CHOP shRNA lentivirus. Supplementary Figure 2: body weight was monitored during the IH treatment. ^∗∗^*P* < 0.01; ^∗∗∗^*P* < 0.001; control group vs. IH group.
