# Supplementary material for: Suppression of CHOP Reduces Neuronal Apoptosis and Rescues Cognitive Impairment Induced by Intermittent Hypoxia by Inhibiting Bax and Bak Activation
Source: Neural Plast. 2021 Aug 21;2021:4090441. doi: 10.1155/2021/4090441 (PMC8405296; doi:10.1155/2021/4090441)
Supplement: Supplementary Materials — Supplementary Figure 1: the expression level of CHOP mRNA was significantly decreased after transfection with CHOP shRNA lentivirus. Supplementary Figure 2: body weight was monitored during the IH treatment. ∗∗P < 0.01; ∗∗∗P < 0.001; control group vs. IH group. [file 4090441.f1.doc]

**Supplementary information**

**Supplementary Methods**

Open-field test of exploratory behavior

An open-field apparatus consisting of a transparent Plexiglas square arena (70×70 cm, 60 cm high) was placed in a homogenously lit experimental room with several large-scale environmental visual cues. Briefly, after 4 weeks of IH exposure, mice were placed in the arena and allowed to explore it for 5 min. During this period, the amount of time animals spent in the three concentric areas was automatically measured as an index of anxious behavior. Since the probability that an animal will explore an area is proportional to the size of the area, the time spent in each area was also divided by the percentage size of the total area to obtain unbiased estimates.

Delay-dependent one-trial object recognition task

Two different objects that differed in height, color, shape, and surface texture were used as previously described. The weights of both objects were sufficiently heavy to ensure that the mice could not displace them, had no ethological significance for the mice, and had never been paired with a reinforcer. Mice were subjected to two types of trials: a sample trial and a test trial. All animals were presented with the first pair of identical objects during the sample trial, which occurred 1 day after exposure to the open field to allow the animals to habituate to the test apparatus. Briefly, the animal was placed on one side of the open field, whereas two copies of the same object were placed 15 cm from the two corners on the opposite side of the apparatus. After 5 min, the animal was removed from the open field and returned to its home cage.

During the test trial, the mice were tested for item recognition (5 min) 2 or 24 h after the sample trial. The animal was again released into the open field, which now contained two objects: a familiar object used during the sample trial and a novel object placed in a random location.

The time each mouse spent exploring the two objects was then recorded. Exploration of an object was recorded when the mouse approached an object and touched it with either the vibrissae, snout, or forepaws. The percentage of time spent exploring the novel object relative to the total time spent exploring both objects was recorded as a measure of object recognition and was calculated using the following formula: recognition index=t_novel_/(t_novel_+t_familiar_)×100. Recognition index values >50 suggest a preference for the novel object, values near 50 suggest no recognition, and values much less than 50 suggest a preference for the familiar object.

Morris water maze test

Spatial learning performance was assessed using the Morris water maze (Electric Factory of Anhui, China) after 2 weeks of IH exposure. A circular, black-painted pool (150 cm in diameter, 50 cm long, and 30 cm deep) was filled with water, which was made opaque by the addition of 30 mL of black ink. An invisible platform (8 cm in diameter) was submerged 1 cm below the water line and placed in the center of the northeast quadrant. The quadrants were labeled north (N), east (E), south (S), and west (W) at equivalent points on the rim. The mice were trained four times per day for four consecutive days. They were allowed a maximum of 120 s to escape onto the hidden platform and were allowed to stay on the platform for 20 s. Rats who failed to locate the platform were placed on it. Each rat was gently placed in the water at one of the starting points with its nose facing the wall. All animals were tested in sequence, and the time intervals between the two trials ranged from approximately 1–1.5 h. Animals were placed at the same starting point each day. The escape latency recorded in four trials per day was averaged. The platform was removed from the pool on day 4, and the average number of attempts required to find the location of the platform within 120 s was recorded.

Isolation of mitochondria from hippocampus

Mitochondria were isolated from hippocampal tissue using the discontinuous Percoll density gradient method. Briefly, brain homogenate was made in ice-cold 12% Percoll solution and centrifuged at 30,700 × g and 4°C for 5 minutes. The upper half of the liquid, which included a layer of material consisting predominantly of myelin, was aspirated to a new tube on ice. The remainder of the centrifuged material in the centrifuge tube was retained on ice. Then, an equal volume of 14% Percoll solution was added to the material removed from the upper portion of the tube, and the material was centrifuged again at 30,700 × g at 4°C for 5 min. The upper half of the centrifuged material was aspirated and discarded. The lower half was pooled with the comparable fraction retained from the ﬁrst centrifugation step. Then, 20 μl of 50 mg/ml digitonin solution was added. Third, the digitonin-treated fraction (up to 3 ml per tube) was then layered with a glass Pasteur pipette on previously prepared discontinuous gradients consisting of 19% Percoll layered over 40% Percoll and centrifuged at 30,700 × g at 4°C for 10 min. Fourth, a glass Pasteur pipette was used to successively remove the upper two layers of the gradient. The Percoll solution containing the enriched mitochondrial fraction (band 3) was then removed to a new tube, and 4 volumes of isolation buffer were added. Finally, after centrifugation at 16,700 × g at 4°C for 10 min, the supernatant was aspirated and discarded. A small amount of material consisting of the mitochondria was left at the base of the tube. After collection, mitochondrial function was immediately detected.

Fluorimetric analysis of mitochondrial membrane potential

Changes in mitochondrial membrane potential were assessed by the lipophilic cationic carbocyanine probe JC-1. Isolated mitochondrial samples were incubated with JC-1 staining buffer according to the manufacturer’s instructions (Sigma, Isolated Mitochondria Staining Kit). The fluorescence intensity of JC-1 aggregates was detected with a 520-nm emission filter, and the JC-1 monomer was measured with 485-nm excitation and 520-nm emission filters using a BMG Novo Star Galaxy spectrofluorometer. The fluorescence intensity ratio of aggregates to monomers was calculated as an indicator of mitochondrial membrane potential.

Construction of the rat CHOP shRNA lentiviral expression vector

CHOP shRNA and scrambled control shRNA were chemically synthesized using Gag/Pol plasmids (Shanghai GenePharma Bio-Tech Company, Shanghai, China), annealed and cloned into the pGag/Pol shRNA lentivector between the HpaI and XhoI sites of the plasmid. Then, the correct insertion of the shRNA cassette was confirmed by direct DNA sequencing. Three plasmids, including the pGag/Pol CHOP shRNA or pGag/Pol control shRNA plasmid, the Rev plasmid and the CMV-VSVG plasmid, were transfected into 293T cells to produce recombinant lentivirus. The culture medium was collected at 72 h posttransfection, concentrated by ultrahigh-speed centrifugation at 26,000 rpm and 4°C for 2 h, aliquoted, and stored at -80°C until use. The titers were determined by fluorescence-activated cell sorting analysis of GFP-positive 293T cells that were transduced with serial dilutions of concentrated lentivirus. The transducing units (TU)/ml were calculated, and the titers were adjusted to 1 × 10^7^ (TU)/ml in this study.

RNA Isolation, Reverse Transcription, and qRT-PCR

PC12 neuron cells were harvested and lysed in TRIzol (Sigma), and then total RNA was extracted. One microgram of RNA was reverse-transcribed into cDNA using SuperScript™ III First-Strand Synthesis SuperMix according to the following procedure: 10 min, 25°C; 30 min, 50°C; and 5 min, 85 °C. The expression levels of CHOP and GAPDH were quantified by Power SYBR® Green PCR Master Mix with the two-step protocol (95°C, 10 min; 40 cycles of 95°C, 15 s and 63°C, 25 s; 95°C, 15 s; 60°C, 1 min). The PCR primers (Invitrogen, USA) used in this study are as follows:

CHOP: forward: 5’-GGGAGCTGGAAGCCTGGTA-3’, reverse: 5’-GCAGGGTCAAGAGTAGTGAAGGT-3’; and

GAPDH: forward: 5’-GAAGGTCGGTGTGAACGGATTTG-3’, reverse: 5’-CATGTAGACCATGTAGTTGAGGTCA-3’.


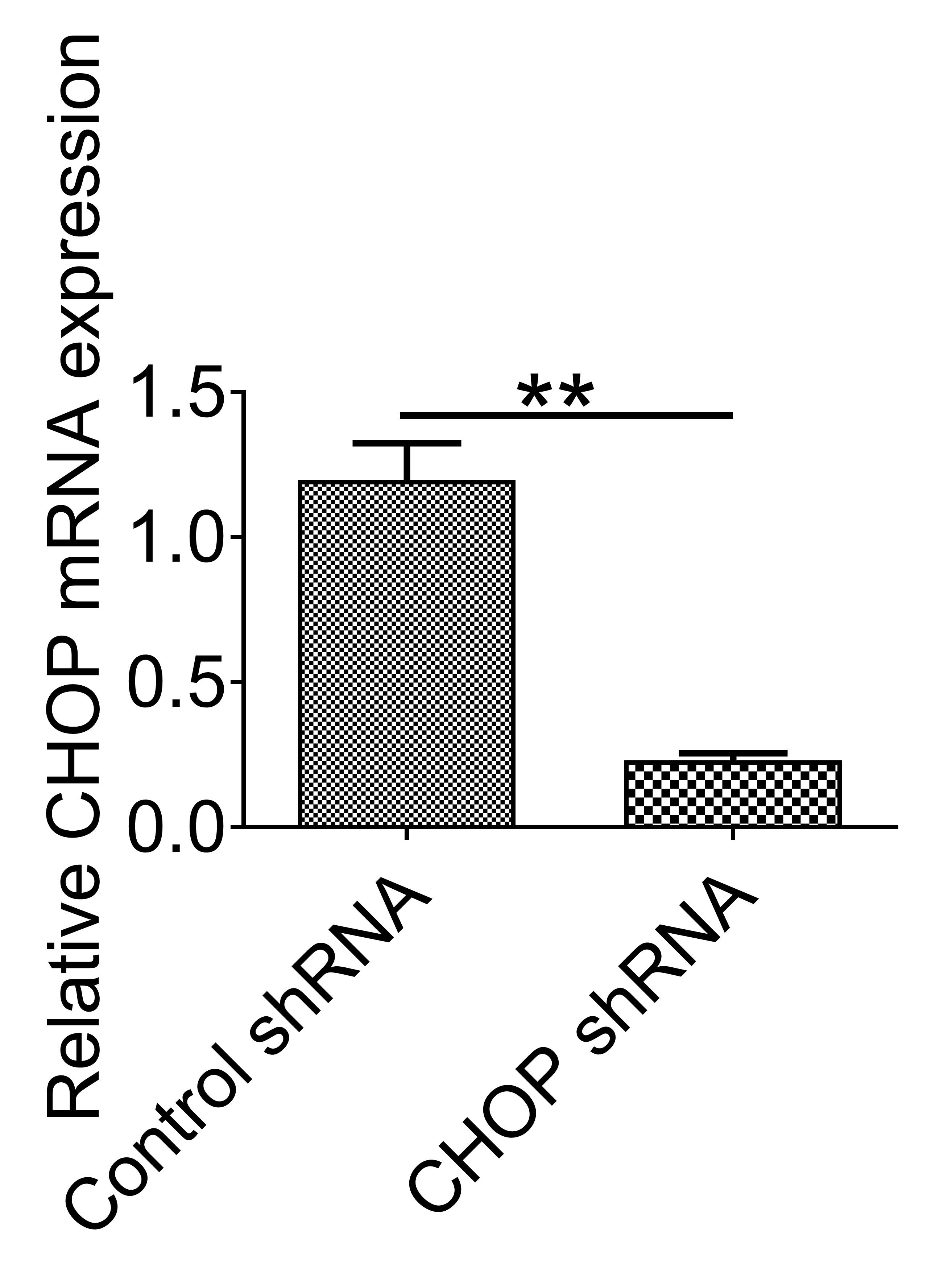
Supplementary Fig 1. The expression level of CHOP mRNA was significantly decreased after transfection with CHOP shRNA lentivirus.


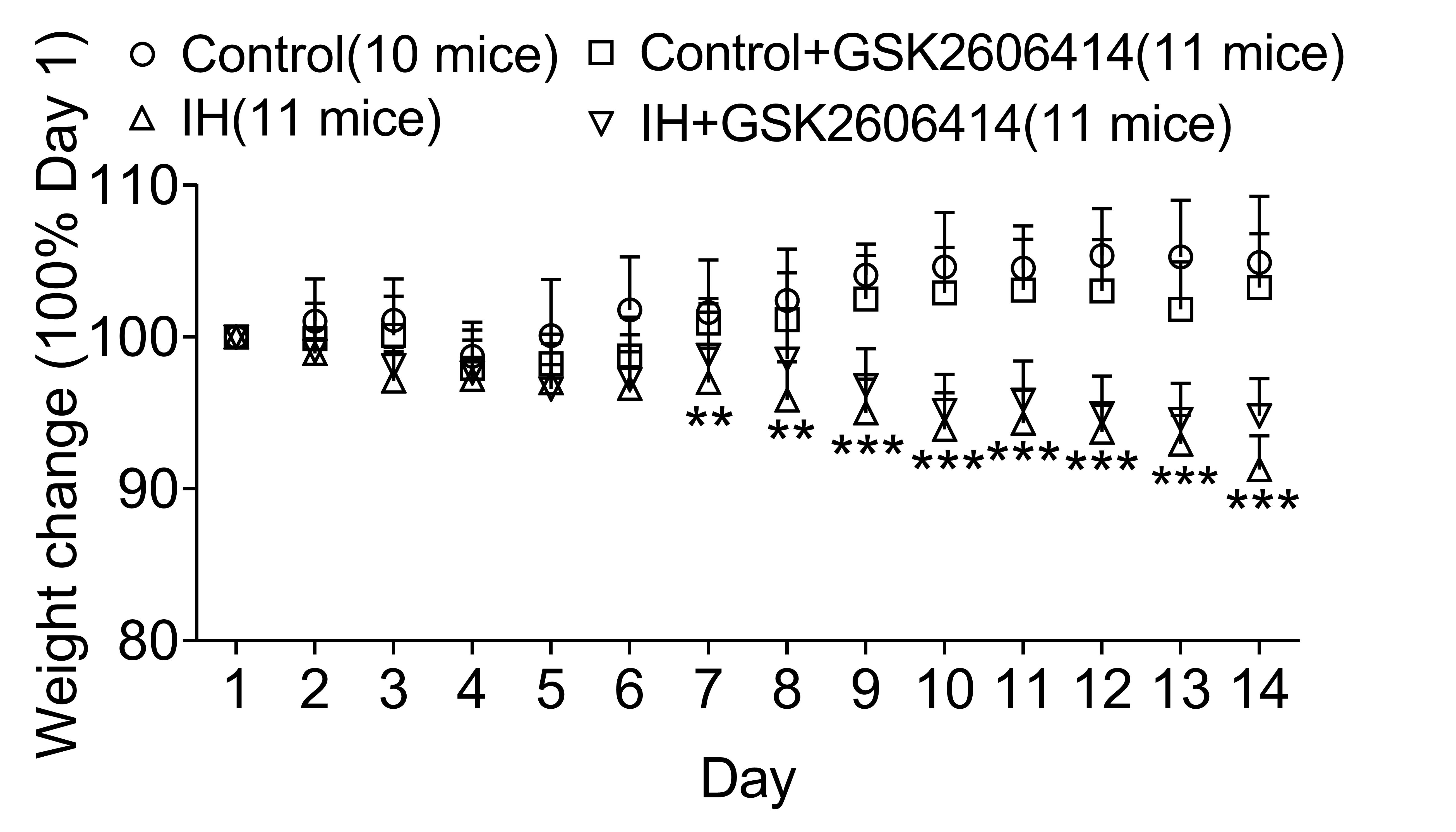
Supplementary Fig 2. Body weight was monitored during the IH treatment. ***P* < 0.01; ****P* < 0.001; control group vs IH group.
